# Histogram analysis of dynamic contrast-enhanced magnetic resonance imaging in the differential diagnosis of parotid tumors

**DOI:** 10.1186/s12880-021-00724-y

**Published:** 2021-12-17

**Authors:** Shiyu Xiang, Jiliang Ren, Zhipeng Xia, Ying Yuan, Xiaofeng Tao

**Affiliations:** Department of Radiology, Shanghai Ninth People’s Hospital, Shanghai Jiao Tong University School of Medicine, 639 Zhizaoju Road, Shanghai, 200011 China

**Keywords:** Parotid tumor, Dynamic contrast-enhanced magnetic resonance imaging, Histogram

## Abstract

**Objective:**

Dynamic contrast-enhanced magnetic resonance imaging (DCE-MRI) histograms were used to investigate whether their parameters can distinguish between benign and malignant parotid gland tumors and further differentiate tumor subgroups.

**Materials and methods:**

A total of 117 patients (32 malignant and 85 benign) who had undergone DCE-MRI for pretreatment evaluation were retrospectively included. Histogram parameters including mean, median, entropy, skewness, kurtosis and 10th, 90th percentiles were calculated from time to peak (TTP) (s), wash in rate (WIR) (l/s), wash out rate (WOR) (l/s), and maximum relative enhancement (MRE) (%) mono-exponential models. The Mann–Whitney U test was used to compare the differences between the benign and malignant groups. The diagnostic value of each significant parameter was determined on Receiver operating characteristic (ROC) analysis. Multivariate stepwise logistic regression analysis was used to identify the independent predictors of the different tumor groups.

**Results:**

For both the benign and malignant groups and the comparisons among the subgroups, the parameters of TTP and MRE showed better performance among the various parameters. WOR can be used as an indicator to distinguish Warthin’s tumors from other tumors. Warthin’s tumors showed significantly lower values on 10th MRE and significantly higher values on skewness TTP and 10th WOR, and the combination of 10th MRE, skewness TTP and 10th WOR showed optimal diagnostic performance (AUC, 0.971) and provided 93.12% sensitivity and 96.70% specificity. After Warthin’s tumors were removed from among the benign tumors, malignant parotid tumors showed significantly lower values on the 10th TTP (AUC, 0.847; sensitivity 90.62%; specificity 69.09%; *P* < 0.05) and higher values on skewness MRE (AUC, 0.777; sensitivity 71.87%; specificity 76.36%; *P* < 0.05).

**Conclusion:**

DCE-MRI histogram parameters, especially TTP and MRE parameters, show promise as effective indicators for identifying and classifying parotid tumors. Entropy TTP and kurtosis MRE were found to be independent differentiating variables for malignant parotid gland tumors. The 10th WOR can be used as an indicator to distinguish Warthin’s tumors from other tumors.

## Introduction

Salivary gland neoplasms constitute approximately 3–5% of all head and neck tumors. Approximately 70% of all salivary gland neoplasms occur in the parotid gland [1, 2]. Accurate differentiation between malignant and benign lesions is important for the determination of therapeutic strategies and the prediction of the disease outcome [3]. Although fine needle biopsy is the gold standard for preoperative diagnosis, it has some shortcomings [4, 5], such as the difficulty of obtaining a definite diagnosis from the biopsy and the accuracy not being ideal in the differential diagnosis of small and/or deep parotid tumors, since salivary gland tumors show various histopathological features [6, 7].

Functional MR imaging techniques have been proven to be useful for quantifying tumor characteristics related to tumor physiology and biology [8, 9]. Dynamic contrast-enhanced magnetic resonance imaging (DCE-MRI) is a functional imaging technique, which is a modality to characterize the perfusion and vascularization of tissues [10, 11]. DCE-MRI has been already proven to provide satisfactory accuracy in differential diagnosis of benign and malignant tumors of the parotid gland [4, 12, 13]. Histogram analysis is a widely used tool for heterogeneity quantification because some MRI features are not always well distinguished with the naked eye [14]. It has been reported that DCE-MRI histogram analysis is effective in differentiating primary central nervous system lymphoma from atypical glioblastoma (GBM) and in detecting local tumor recurrence after treatment of head and neck squamous cell carcinoma [15, 16].

In the field of parotid tumor imaging, histogram analysis of apparent diffusion coefficient (ADC) maps has already been used to differentiate malignant from benign parotid tumors [17, 18]. However, to the best of our knowledge, the Whole-volume tumor DCE-MRI histogram analysis for differentiating malignant from benign parotid gland tumors has not been reported till now. Even some DCE-MRI parameters, such as maximum relative enhancement (MRE) (%), time to peak (TTP) (s), wash in rate (WIR) (l/s) and wash out rate (WOR) (l/s) have barely been studied.

Whole-volume DCE-MRI histograms were used in this study to analyze parotid gland tumors. By adopting whole-volume DCE-MRI measurements, the sampling error of the selected region of interest (ROI) can be minimized.

The purpose of this study was to verify the value of DCE-MRI histogram analysis with the above parameters in differentiating benign and malignant tumors and further differentiating the tumor subgroups.

## Materials and methods

### Patients

The institutional review committee of Shanghai Ninth People's Hospital approved the retrospective study and exempted the requirement of informed consent. Patients with parotid gland tumors who were admitted and treated in our hospital between January 2018 and June 2020 were included in the study. All patients underwent DCE-MRI examination for pre-surgery evaluation of parotid gland tumors. According to the following exclusion criteria: (1) no DCE-MRI; (2) with biopsy before examination; (3) residual disease after treatment; (4) simple cystic disease; (5) tumor short axis diameter less than 1 cm; (6) there are serious motion artifacts. Finally, 117 patients were enrolled in this study (32 malignant and 85 benign). 57 women and 60 men aged between 9 and 85 years (mean 47.1 ± 16.9 years) were including in the study group. All of these tumors were diagnosed via pathological examinations after surgery. The criteria for collecting cases were similar to the previous histogram research methods [19].

### MRI protocol

MRI examinations were performed with a 3.0 T scanner (Philips Ingenia 3.0 T; Amsterdam, the Netherlands). All examinations consisted of a 5-mm section thickness and a 1-mm intersection gap, 264 × 239 acquisition matrix, and 210 × 192 mm field of view (FOV). The transverse T1-weighted repetition time (TR) and echo time (TE) were 479 ms and 12 ms, respectively. Transverse T2-weighted fast spin echo (FSE) sequences were performed with a TR of 3800 ms and a TE of 70 ms. FSE T1-weighted images were obtained with a TR of 400 ms and a TE of 9 ms. Gadolinium diethylenetriamine pentaacetic acid (Gd-DTPA; Magnevist, Schering, Berlin, Germany) was administered intravenously at a rate of 2–3 ml/s (total dose 0.1 mmol per kg of body weight), followed by a 20-ml saline flush. 32 phases were collected by dynamic enhancement sequence.

### Imaging analysis

**Fig. 1:**
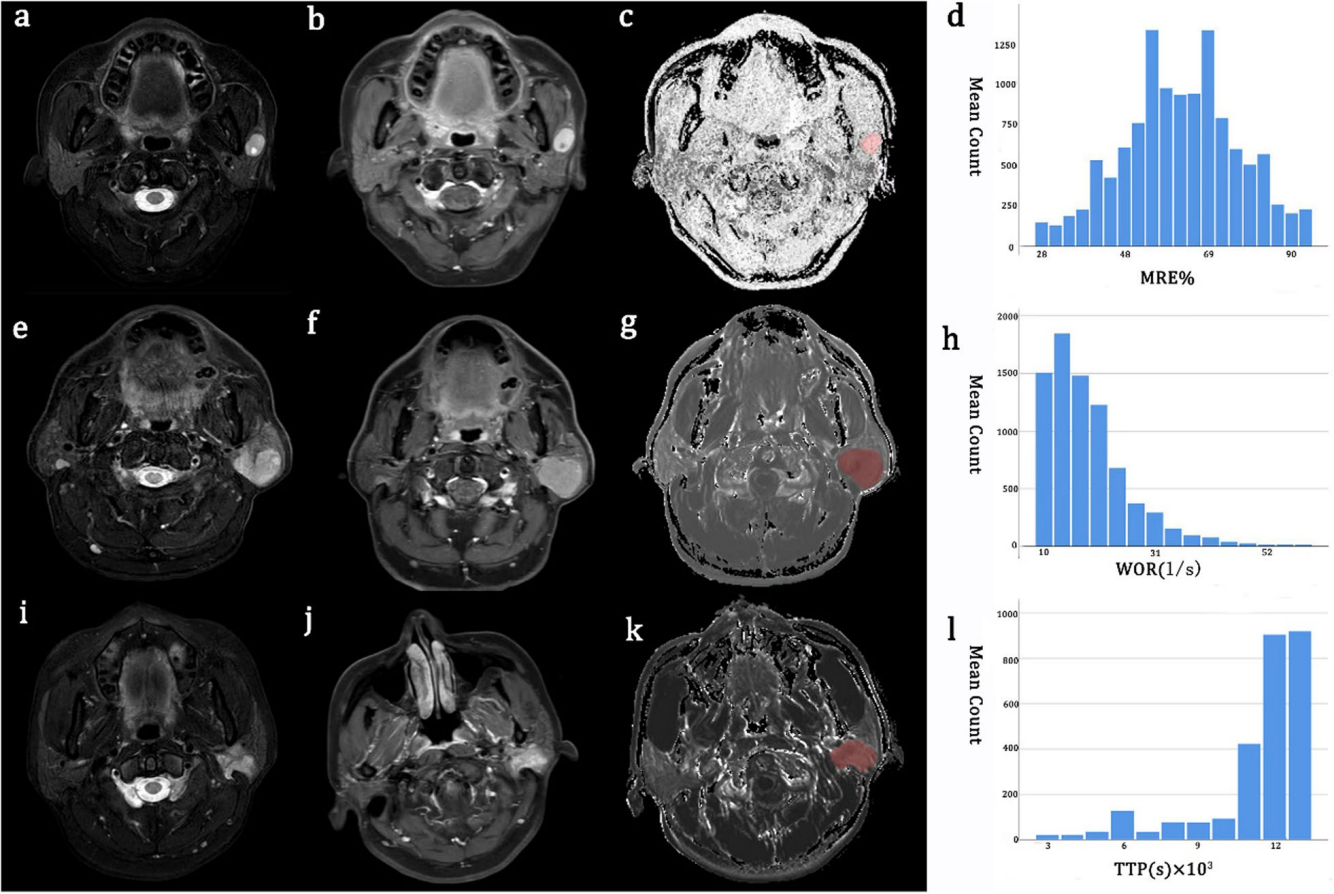
Representative images of a 57-year-old woman with pleomorphic adenoma (**a**–**d**), a 56-year-old man with Warthin’s tumor (**e**–**h**) and a 43-year-old woman with mucoepidermoid carcinoma (**i**–**l**). The left column was the axial T2-weighted image with fat suppression (**a**, **e**, **i**), the middle column was the axial T1-weighted enhancement image (**b**, **f**, **j**). After the ROIs were placed (**c**, **g**, **k**), corresponding histogram maps showed a high value MRE of pleomorphic adenoma (**d**), a low WOR value of Warthin’s tumor (**h**), and a high value TTP of mucoepidermoid carcinoma (**l**)

We generated different parameter maps of DCE-MRI through a Philips postprocessing workstation, including TTP, WIR, WOR, and MRE. The ROIs were independently drew on MRE manually through ITK-SNAP software by two radiologists (with seven and three years of experience in head and neck radiology, respectively), with contrast-enhanced T1WI images used as a reference. They were blinded to the clinical information and pathological results of patients. A series of ROIs were manually drawn to cover the lesion as completely as possible on each slice of the mass (Fig. 1). The ROI was carefully placed to exclude necrotic, calcified areas and blood vessels. Regarding bilateral lesions, we chose lesions with larger diameters for analysis. Some ROI sketching methods learned from previous histogram studies of parotid tumor [18]. The ROIs created a 3D tumor model and then copied it to every parameter diagram automatically, including WIR, WOR, and TTP. For each DCE–MRI parameter map, the following data were extracted by 3D Slicer software: mean and median values, as well as 10th and 90th percentiles, were estimated. In addition, histogram-based characteristics, kurtosis, skewness, and entropy, were also calculated.

### Statistical analysis

Normal distribution of continuous variables was verified with the Kolmogorov–Smirnov test. Normally distributed data were presented as the mean difference ± standard deviation. Data with a abnormal distribution were reported as the median ± interquartile range. Significance of intergroup differences in the characteristics of continuous variables was verified with Student t-test or Mann–Whitney U test, and Pearson’s chi-square test or Fisher exact test were used for intergroup comparisons of discrete variable distributions. ROC curve analysis was performed on the mean value of the histogram to obtain the cutoff value, area under the curve, sensitivity, and specificity. Then, parameters that were shown to have univariate associations with malignant parotid tumors were included in a multivariable stepwise logistic regression analysis to identify the independent predictors of the malignant parotid tumors.

The two-way intraclass correlation coefficient (ICC) was used to assess interobserver agreement for the parameters. According to previous research [19, 20], the ICC value > 0.81 was considered as almost perfect agreement. All calculations were carried out with statistical packages (SPSS Version 25.0 and MedCalc version 11.0), with the threshold of statistical significance set at *P* < 0.05.

## Results

No significant difference was observed for patient age or sex between the two groups (Table 1). Interobserver agreement in the DCE-MRI analysis was perfect for all pharmacokinetic parameters (ICCs, range = 0.920–0.976). Representative cases of pleomorphic adenoma, Warthin’s tumor and mucoepidermoid carcinoma are presented in Fig. 1.

**Table 1:** Summary of the demographic and pathological characteristics of the parotid gland tumors

Parameters	Benign tumors (n = 85)	Malignant tumors (n = 32)	*P* value
Mean age (years)	48.4 ± 16.2	43.8 ± 18.4	0.185
Sex (M/F)	44/41	16/16	0.865
	Pleomorphic adenomas (45)	Mucoepidermoid carcinoma (12)	
	Warthin’s tumors (32)	Acinic cell carcinoma (6)	
	Base cell adenoma (8)	Marginal zone B-cell lymphoma (4)	
	Pleomorphic adenomas (2)	Adenoid cystic carcinoma (3)	
		Adenocarcinoma (2)	
		Squamous cell carcinoma (2)	
		Carcinoma ex pleomorphic adenoma (2)	
		Lymphoepithelial carcinoma (1)	

Age is reported as the mean ± standard deviation. Numbers in parentheses denote the number of people

## Differential diagnostics of parotid benign and malignant tumors

DCE-MRI-derived histogram parameters in both groups are summarized in Table 2. For the TTP-related histogram parameters, the 10th TTP (*P* = 0.017) and kurtosis (*P* = 0.003) were significantly higher in the benign parotid tumor group than in the malignant parotid tumor group, while entropy (*P* < 0.000) was significantly lower in the benign tumor group than in the malignancy group. For MRE-related histogram parameters, kurtosis (*P* < 0.000) was significantly higher in the malignant parotid tumor group than in the benign parotid tumor group, while skewness (*P* = 0.002) was significantly higher in the benign parotid tumor group than in the malignant parotid tumor group. No significant differences were found in any WIR, or WOR-related histogram parameters between the two groups (all *P* values > 0.05).

**Table 2:** Histogram parameters of the benign and malignant parotid gland tumor groups

Parameters	Benign parotid tumors (n = 85)	Malignant parotid tumors (n = 32)	*P* value
Histogram of TTP (s)			
10th TTP	80.231 ± 34.760	62.655 ± 20.500	0.017
Mean TTP	101.542 ± 34.012	93.901 ± 21.753	0.290
Median TTP	99.441 ± 65.624	94.399 ± 30.906	0.266
90th TTP	120.067 ± 34.311	123.310 ± 33.052	0.966
Kurtosis	5.929 ± 6.218	3.408 ± 2.475	0.003
Skewness	0.437 ± 1.613	0.545 ± 0.940	0.565
Entropy	1.344 ± 0.620	1.467 ± 0.350	0.000
Histogram of WIR (l/s)			
10th WIR	40.012 ± 10.110	40.002 ± 20.001	0.835
Mean WIR	50.340 ± 20.536	60.023 ± 20.534	0.727
Median WIR	52.080 ± 27.573	54.963 ± 19.311	0.755
90th WIR	70.431 ± 30.682	80.603 ± 30.713	0.802
Kurtosis	3.723 ± 1.423	4.548 ± 3.133	0.160
Skewness	0.543 ± 0.448	0.729 ± 0.748	0.134
Entropy	1.328 ± 0.406	1.401 ± 0.447	0.409
Histogram of WOR (l/s)			
10th WOR	4.942 ± 7.070	3.165 ± 4.852	0.112
Mean WOR	18.345 ± 11.465	20.007 ± 10.112	0.255
Median WOR	14.804 ± 14.456	16.471 ± 9.283	0.255
90th WIR	34.726 ± 22.734	37.812 ± 20.054	0.219
Kurtosis	4.710 ± 4.221	4.460 ± 1.327	0.322
Skewness	0.917 ± 0.675	0.774 ± 0.485	0.427
Entropy	1.249 ± 0.467	1.346 ± 0.607	0.221
Histogram of MRE (%)			
10th MRE	86.363 ± 24.673	83.128 ± 26.243	0.718
Mean MRE	113.712 ± 32.043	111.047 ± 34.471	0.922
Median MRE	106.587 ± 47.462	107.831 ± 39.139	0.835
90th MRE	143.807 ± 46.021	140.212 ± 46.215	0.874
Kurtosis	9.237 ± 13.233	29.510 ± 53.624	< 0.000
Skewness	0.749 ± 0.730	0.728 ± 0.730	0.002
Entropy	1.854 ± 0.495	1.872 ± 0.571	0.448

Except for *P* values, data are reported as the median ± interquartile range

**Fig. 2:**
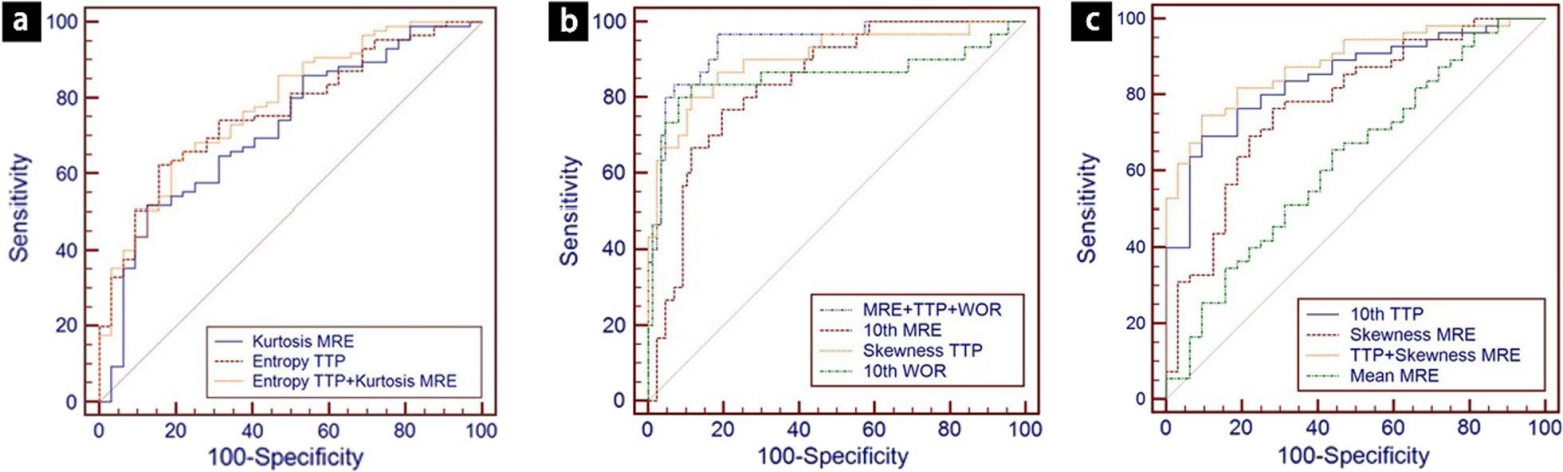
ROC curve of metrics performed in different groups analysis of parotid tumors. **a** ROC curves illustrated the accuracy of MRI with cutoff values entropy TTP > 3.21 and kurtosis MRE > 4.47 alone and combined them in the differential diagnostics of parotid malignancies and benign tumors. **b** ROC curves illustrated the accuracy of MRI with cutoff values of 10th MRE < 75.67%, skewness TTP ≥ − 0.06 and 10th WOR ≥ 7.35 l/s alone combined them in the differential diagnostics of Warthin’s tumors from other tumors. **c** ROC curves illustrated the accuracy of MRI with cutoff values 10th TTP < 84.92 s and skewness MRE ≥ 1.00 in the differential diagnostics of parotid malignancies and benign tumors except Warthin’s tumors

Table 3 summarizes the multivariable stepwise logistic regression analysis results for identifying the independent predictors of malignancy. With a cutoff value of 3.21, the entropy of TTP showed an optimal diagnostic performance (area under the curve = 0.762, sensitivity = 84.37%, specificity = 62.35%) for differentiating between parotid benign and malignant tumors. The ROC analysis results also showed that when the kurtosis MRE set mean was ≤ 4.47, the area under the ROC curve was 0.720, and the sensitivity and specificity were 87.50% and 51.76%, respectively, in discriminating between parotid benign and malignant tumors (*P* = 0.0248). Including these two variables in the ROC model resulted in further improvement of the diagnostic accuracy of MRI (Fig. 2a).

**Table 3:** ROC curve analyses of the significant histogram parameters for differentiating malignant from benign parotid tumors

Parameters	Benign parotid tumors	Malignant parotid tumors	*P* value	AUC (95% CI)	Cut off value	Sensitivity Specificity
Entropy TTP	1.344 ± 0.620	1.467 ± 0.350	< 0.000	0.762 (0.675–0.836)	> 3.21	84.37% 62.35%
Kurtosis MRE	9.237 ± 13.233	29.512 ± 53.631	0.025	0.720 (0.629–0.799)	> 4.47	87.50% 51.76%
TTP + MRE			< 0.000	0.781 (0.695–0.852)		81.35% 63.53%

## Multiple comparisons among subgroups

### Warthin’s tumors vs other tumors

Compared to other tumors, Warthin’s tumors showed significantly lower values on 10th MRE and significantly higher values on skewness TTP and 10th WOR (Table 4). ROC analysis confirmed that the cutoff values that differentiated between Warthin’s tumors and other lesions with an optimum accuracy were 10th MRE < 75.67%, skewness TTP ≥  − 0.06 and 10th WOR ≥ 7.35 l/s. Consideration of all three cutoff values resulted in improvement of the accuracy of MRI in differential diagnosis of the two types of lesions (Fig. 2b). The sensitivity and specificity of the combined diagnostic model were 96.70% and 93.12%, respectively.

**Table 4:** ROC curve analyses of the significant histogram parameters for differentiating Warthin tumors from the other tumors

Parameters	Warthin’s tumors	Tumors except Warthin’s tumor	*P* value	AUC (95% CI)	Cut off value	Sensitivity Specificity
10th MRE (%)	62.047 ± 27.212	92.436 ± 27.449	< 0.001	0.838 (0.759–0.900)	< 75.67	86.67% 80.46%
Skewness TTP	0.937 ± 1.826	− 1.002 ± 1.208	< 0.001	0.903 (0.834–0.950)	≥ − 0.06	80.00% 88.51%
10th WOR (l/s)	12.166 ± 9.624	− 0.006 ± 0.582	< 0.001	0.855 (0.778–0.913)	≥ 7.35	80.00% 91.95%
MRE + TTP + WOR				0.971 (0.922–0.993)		96.70% 93.12%

## Malignant tumors vs benign tumors except Warthin tumors

After the Warthin’s tumors were removed from the benign tumors, the remaining benign tumors were compared with malignant parotid tumors. Malignant parotid tumors show significantly lower values on 10th TTP and mean MRE than the benign parotid tumors, while malignant parotid tumors show higher values in skewness MRE than the benign parotid tumors. The detailed parameters differentiating between the benign tumors and the malignant parotid gland tumors are summarized in Table 5. Considering the 10th TTP and the mean MRE, these two variables in the ROC model resulted in further improvement of the diagnostic accuracy of MRI (Fig. 2c).

**Table 5:** ROC curve analyses of the significant histogram parameters for differentiating malignant from benign parotid tumors except Warthin tumors

Parameters	Malignant tumors	Benign tumors except Warthin’s tumor	*P* value	AUC (95% CI)	Cut off value	Sensitivity Specificity
10th TTP (s)	62.341 ± 29.120	94.511 ± 41.233	< 0.001	0.847 (0.753–0.915)	< 84.92	90.62% 69.09%
Mean MRE (%)	111.021 ± 34.352	127.501 ± 28.645	0.040	0.628 (0.518–0.730)	< 116.83	86.25% 65.45%
Skewness MRE	1.697 ± 1.799	0.382 ± 0.853	0.005	0.777 (0.675–0.859)	≥ 1.00	71.87% 76.36%
TTP + Skewness MRE				0.885 (0.798–0.943)		90.62% 74.55%

From above, we can draw a recursive partition for parotid gland tumor based on histogram analysis, which may help us diagnose in clinical work. We hypothesize we can distinguish Warthin’s tumors based on MRE, TTP and WOR, especially WOR. Then, 10th TTP and Skewness TTP may help us better distinguish the remaining benign tumors from malignant tumors. As it should be, the idea of this recursive partition needs to be verified in future research.

## Discussion

In this study, we investigated the predictive value of DCE-MRI-derived histogram parameters in differentiating malignant from benign parotid tumors. DCE-MRI provides a noninvasive assessment of tumor perfusion and vascularity, has been widely applied in the field of parotid gland imaging [12, 21].

The most commonly diagnosed histological types of benign parotid lesions are pleomorphic adenomas and Warthin’s tumors [2]. According to the literature, pleomorphic adenomas are characterized by a gradual enhancement (high TTP) and subsequent slow contrast washout (low WOR) [4, 22] and Warthin’s tumors and malignant lesions show early enhancement (low TTP), followed by rapid (high WOR) [8, 12, 23] or gradual washout (low WOR) [24], which may interfere with the differentiation of benign and malignant parotid tumors.

In our study, we also found TTP parameters can be used as an identification point, and MRE was also included. In further analysis using multivariate stepwise logistic regression analysis, entropy TTP and kurtosis MRE were found to be independent differentiating variables for malignant parotid gland tumors. When using kurtosis MRE alone for diagnosis, the sensitivity can reach 87.50%. Entropy is one of the parameters that characterizes the state of matter in thermodynamics. The smaller the entropy is, the more orderly it is. Also, the larger the entropy is, the more disordered it is [25, 26]. We believe that the blood vessels and cells in benign tumors are arranged more orderly. On the contrary, the tissue structure in malignant tumors is more chaotic and blood vessels are immature. Therefore, the Entropy TTP value was lower in benign parotid gland tumors. Kurtosis is a statistical parameter to describe the shape of the data distribution [27]. The lower kurtosis MRE of benign tumors indicates that the cells are more uniform than in malignant tumors and that the DCE-MRI parameters are concentrated.

In the comparison among subgroups, the TTP and MRE parameters still showed better performance among the various pharmacokinetic parameters. In addition, we found that Warthin’s tumors showed significantly higher values in the 10th WOR than the other tumors. In previous studies, some scholars found that the WOR for Warthin’s tumors was significantly higher than that for malignant tumors [12]. Meanwhile, in other studies, some researchers reported that all Warthin’s tumor lesions could be successfully differentiated from pleomorphic adenomas and malignant tumors by using WOR [4]. Therefore, we may use WOR as an indicator to distinguish Warthin’s tumors from the other tumors. When combining 10th MRE, skewness TTP and 10th WOR, this resulted in a marked improvement in the diagnostic accuracy (93.12% sensitivity and 96.70% specificity). Gökçe, E [13] summarized the research of using dynamic enhanced MRI parameters to identify Warthin’s tumors in recent ten years. In this study, the sensitivity ranged from 71.4% to 100%, and specificity ranged from to 91.2%-100%. Compared with our research results, our study has relatively high sensitivity and specificity. Mikaszewski B et al. used some DCE-MRI parameters, such as T_peak_, T_max_ to differentiate between parotid malignancies, pleomorphic adenomas and Warthin’s tumors. The highest sensitivity and specificity were 84.2% and 91.9%. They combined T_peak_, WR, ADC value in diagnosis Warthin’s tumor from malignant tumor, which the sensitivity and specificity were lower than our study [28]. TTP is known to correlate inversely with microvessel density in the examined tissue, and WOR was shown to increase proportionally to the amount of connective tissue within the lesion [29]. The skewness reflects the asymmetry of the distribution of parameters. The skewness of the Warthin’s tumor group is greater than that of the other tumor group, indicating that there are more extreme values at the right end of the data, which shows that the TTP signal of the Warthin’s tumor tends to be concentrated in the low TTP value region.

After the Warthin’s tumors were removed from benign tumors, TTP and MRE still showed good performance. The malignant parotid tumor group showed a significantly lower value on 10th TTP and a higher value on skewness MRE than the benign tumor group except for the Warthin’s tumor group. Compared with previous studies [9, 28], most of them compared pleomorphic adenoma with malignant tumor, whether ADC histogram or dynamic enhanced MRI parameters were used. However, ADC_mean_, T_peak_ which commonly used in clinic does not show good sensitivity and specificity. DCE-MRI histogram shows better discrimination ability in differential diagnostics of parotid benign tumor (excluding Warthin’s tumor) and malignant tumors, which had a higher sensitivity in MRE and TTP. Skewness is also a general indicator of tumor heterogeneity, which reflects the asymmetry of the distribution of DCE-MRI parameters [27]. The skewness of the malignant group is greater than that of the benign group, indicating that there are more extreme values at the right end of the data, which shows that the MRE signal of the malignant group tends to be concentrated in the high MRE value region.

It must be mentioned that all functional imaging techniques, including DCE-MRI histograms, cannot diagnose diseases independently [30]. Some studies have combined DCE-MRI parameters with other magnetic resonance techniques, such as ADC values, and found that combined applications can improve diagnostic accuracy [31]. The DCE-MRI histogram used in this study is a method of texture analysis (TA). In the future, we will contact engineers who specialize in medical imaging to explore the application of radiomics in parotid tumors.

Our study had several limitations. First, it is a retrospective study with relatively few cases in each tumor group. We will continue to collect more patients and expand the sample size to strengthen the statistical power and confirm our results. Second, despite using the whole-volume ROI, the placement of the ROIs was still performed in a manual pattern, which is prone to measurement variability. It has been reported that semiautomatic lesion segmentation could reduce variability and improve reproducibility [32]. The strategy of outlining ROI should be further optimized in future research.

## Conclusion

DCE-MRI histogram is an effective tool for identifying benign and malignant tumors of the parotid gland as well as distinguishing different types of parotid tumors. The TTP and MRE parameters showed good performance in the differential diagnosis of parotid tumors. Entropy TTP and kurtosis MRE were found to be independent differentiating variables for malignant parotid gland tumors, while 10th WOR may be used as an indicator to distinguish Warthin’s tumors.

## Data Availability

The authors declare that all data supporting the findings of this study are available within the paper and its source data for the figures in this study are available from the authors upon request.
